# Enzymatic and microbial routes to bioplastics: The green chemistry frontier of biopolymers

**DOI:** 10.1002/2211-5463.70241

**Published:** 2026-03-27

**Authors:** Giovanni Gallo, Emma Piccoli, Luca Bombardi, Martina Aulitto, Salvatore Fusco

**Affiliations:** ^1^ Biochemistry and Industrial Biotechnology (BIB) Laboratory, Department of Biotechnology University of Verona Italy; ^2^ Department of Agricultural Sciences University of Naples Federico II Portici Italy; ^3^ Department of Biology, University of Napoli Federico II Complesso Universitario Monte Sant'Angelo Naples Italy

**Keywords:** bioplastic copolymers, enzymatic polymerisation, metabolic engineering, PHA synthase

## Abstract

The rapid escalation of global plastic consumption, coupled with the environmental impacts of petrochemical polymers, has sparked a surge of interest in bioplastics, particularly those derived from microbial and enzymatic processes. This review provides a comprehensive overview of the metabolic pathways, structural properties and emerging technological innovations shaping the next generation of bioplastics, with a particular focus on polyhydroxyalkanoates (PHA). The following sections outline the conceptual distinctions between bio‐based and biodegradable plastics, the key bacterial pathways responsible for the biosynthesis of PHA, PLA precursors, bacterial cellulose, microbial polyamides and other bio‐derived polymers. The physicochemical and morphological features of PHA‐based materials are analysed as well. These features include monomer composition, crystallinity, copolymer architecture and molecular weight. The relationship between these features and the mechanical and thermal performance of the materials is then investigated. A dedicated section is allocated to recent advances in *in vitro* enzymatic PHA synthesis, covering PHA synthase (PhaC) classes, engineered variants, cell‐free metabolic engineering platforms, enzyme immobilisation and surface‐display strategies that enable fully programmable and modular polymerisation. Finally, we discuss future perspectives, with particular emphasis on sustainable feedstocks, process intensification through synthetic biology, techno‐economic challenges and the regulatory landscape required for large‐scale adoption. The present review integrates biochemical, structural and bioprocessing insights to map current progress and identify strategic directions for enabling enzymatic bioplastics as scalable, customisable and environmentally sound alternatives within a circular bioeconomy framework.

**Impact statement**

This review highlights recent advances in microbial and enzymatic routes for producing polyhydroxyalkanoate‐based bioplastics, with emphasis on engineered enzymes and cell‐free systems. By integrating biochemical and bioprocess insights, it outlines strategies to enable scalable and sustainable biopolymer production within a circular bioeconomy.

## Graphical abstract

Microbial biosynthesis and engineered enzyme platforms are expanding the design space of polyhydroxyalkanoate bioplastics. By combining fermentation, PHA synthase engineering and cell‐free modular systems, tailored biodegradable polymers can be produced with tunable properties, supporting more sustainable materials and future circular bioeconomy strategies. Created in biorender. Fusco, S. (2026) https://BioRender.com/kvvewnr.

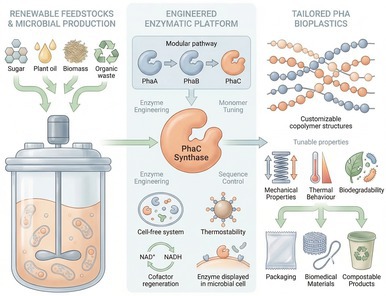

**Abbreviations**
3HV: 3‐hydroxyvalerate
3HB: 3‐hydroxybutyrate
3HH: 3‐hydroxyhexanoate
3HHx: 3‐hydroxyhexanoate unit
BDO: 1,4‐butanediol
CFME: cell‐free metabolic engineering
CoA: coenzyme A
FDCA: 2,5‐furandicarboxylic acid
GRAS: Generally Recognised As Safe
HA hydroxyacyl: LCA life‐cycle analysis
LA: lactate
LCL‐PHA: long‐chain‐length polyhydroxyalkanoate
LPE: lactate‐polymerising enzyme, medium‐chain‐length polyhydroxyalkanoate
Mw: molecular weight
P(3HB‐4HB)‐3HV: poly(3‐hydroxybutyrate‐co‐4‐hydroxybutyrate‐co‐3‐hydroxyvalerate)
P(3HB‐co‐3HV): poly(3‐hydroxybutyrate‐co‐3‐hydroxyvalerate)
P(3HB‐co‐3HHx): poly(3‐hydroxybutyrate‐co‐3‐hydroxyhexanoate)
PBAT: poly(butylene adipate‐co‐terephthalate)
PBS: poly(butylene succinate) PCL polycaprolactone
PE: polyethylene
PEF: poly(ethylene furanoate)
PET: poly(ethylene terephthalate)
PHA: polyhydroxyalkanoate
PhaC: PHA synthase
PHB: poly(3‐hydroxybutyrate)
PHBH: poly(3‐hydroxybutyrate‐co‐3‐hydroxyhexanoate)
PHBV: poly(3‐hydroxybutyrate‐co‐3‐hydroxyvalerate)
PLA: polylactic acid
PP: polypropylene
SCL‐PHA: short‐chain‐length polyhydroxyalkanoate
SpyTag/SpyCatcher: molecular tagging/scaffolding system
Tg: glass transition temperature
Tm: melting temperature
γ‐PGA: poly‐γ‐glutamic acid
ε‐PL: epsilon‐poly‐L‐lysine

## Bio‐based and biodegradable plastics: concepts and distinctions

**Fig. 1:**
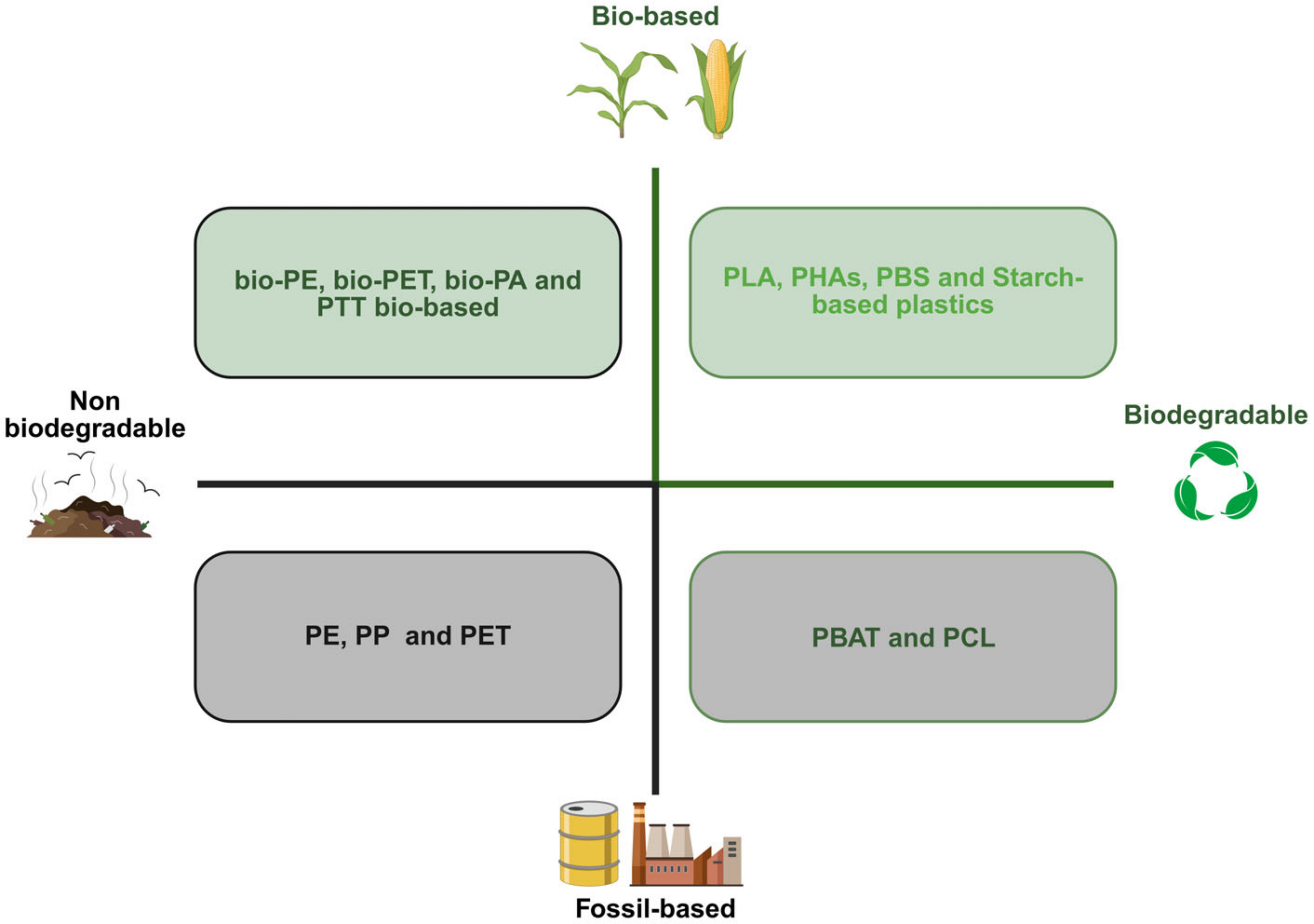
Classification of plastics by origin and biodegradability. The diagram distinguishes four categories of plastics by crossing origin (bio‐based vs fossil‐based) and biodegradability. Bio‐based but nonbiodegradable plastics, for example bio‐PE (polyethylene) and bio‐PET (polyethylene terephthalate) are produced from renewable feedstocks but are chemically identical to fossil‐derived polymers. Bio‐based and biodegradable plastics, for example PLA (polylactic acid), PHA (polyhydroxyalkanoate), PBS (polybutylene succinate), starch‐based materials combine renewable sourcing with microbial degradability. Fossil‐based, nonbiodegradable plastics, for example PE, PP (polypropylene), PET), represent traditional polymers with persistent environmental impact. Fossil‐based but biodegradable plastics, for example PBAT (polybutylene adipate‐co‐terephthalate), PCL (polycaprolactone) show that biodegradability does not depend on bio‐origin. The figure clarifies that ‘bio‐based’ and ‘biodegradable’ are independent attributes.

The definition of bioplastic encompasses polymeric materials that may be bio‐based and/or biodegradable. Specifically, a plastic material is designated as a bioplastic if it is produced (even partially) from renewable biomass or if it is susceptible to biodegradation by microorganisms (or exhibits both properties) [1]. It is imperative to acknowledge the distinction between the concepts of bio‐based and biodegradable, as these two terms do not imply one another. It must be clarified that not all plastics derived from renewable sources are biodegradable, and conversely, not all biodegradable plastics are derived from biomass [2]. For instance, there are bio‐based polymers that are chemically identical to petrol‐based plastics (e.g. polyethylene or PET obtained from biological resources), as well as synthetic polymers of fossil origin designed to be biodegradable (e.g. polybutylene adipate‐co‐terephthalate, PBAT). The prefix ‘bio’ thus indicates the renewable nature of the raw material utilised for the production, without assuring that the resulting polymeric material is intrinsic biodegradable. The term ‘bioplastics’ encompasses a heterogeneous family of polymers with various combinations of raw material sources and end‐of‐life treatments (Fig. 1). The impetus for the development of bioplastics is twofold, with environmental and industrial motivations underpinning this field of research. The objective is twofold: first, to mitigate the impact of conventional plastics; and second, to make this materials' industrial sector more environmentally sustainable. The global production of traditional plastics has now reached approximately 400 million tonnes per year. However, less than 10% of this volume is recycled; the remainder accumulates in landfills or disperses into terrestrial and marine ecosystems, thereby contributing to widespread pollution from plastic waste and microplastics [3]. This critical environmental issue, in conjunction with the exhaustion of fossil resources, has prompted the exploration of more sustainable alternatives. It is hypothesised that bio‐based polymers have a lower carbon footprint than petrochemical plastics. This is because the CO_2_ emitted during their production and degradation can be offset by the CO_2_ absorbed by plants during the growth of the biomass used. In summary, the utilisation of renewable raw materials facilitates the partial closure of the carbon cycle, thereby integrating plastics production into a circular economy paradigm. The growing public awareness of environmental issues, coupled with increasingly stringent environmental regulations (e.g. bans on single‐use plastics and recycling targets), has also encouraged the adoption of bioplastics to reduce dependence on fossil fuels and the environmental impact of the plastics cycle. As of 2022, the global production of biodegradable bioplastics had reached approximately 2.2 million tonnes, and it is projected to triple by 2027. This reflects a surge in industrial investment and regulatory pressure towards sustainable materials [4, 5]. In the context of this rapidly expanding sector, polyhydroxyalkanoates (PHA) constitute a minor yet growing segment. Despite accounting for a limited share of global output, PHA offer unique advantages, including complete biodegradability, biocompatibility and microbial derivation, which renders them particularly suited for high‐value applications, such as packaging, agriculture and biomedical devices [6]. The increasing number of industrial‐scale facilities producing PHA, as well as the entrance of multinational actors, signal that this class of polymers is gaining economic traction. Concurrently, national and supranational policy frameworks are fostering favourable conditions for the adoption of biodegradable and bio‐based plastics [7]. For instance, the European Union's Single‐Use Plastics Directive prohibits the use of several fossil‐based disposable items, and recent legislative proposals advocate for the utilisation of certified compostable packaging in specific applications. Concurrently, jurisdictions such as California and Canada have implemented packaging regulations that explicitly encourage the use of compostable alternatives [5]. The combination of these measures with the increasing alignment of composability standards (e.g. EN 13432, ASTM D6400) and heightened public awareness of plastic pollution is precipitating a shift towards PHA and other enzymatically derived polymers as viable, scalable solutions in a circular bioeconomy [8].

Bioplastics are promoted as ‘green’ alternatives to traditional plastics, with the potential to contribute to sustainability [4]. However, their practical use still requires adequate end‐of‐life management systems and compliance with circularity criteria in material design. In this context, the development of new polymers and production processes with a focus on sustainability is driving innovation in the chemical industry towards products with a reduced environmental impact.

From a technical standpoint, bioplastics can be categorised into two broad classifications: biodegradable polymers (which are frequently bio‐based) and nonbiodegradable bio‐based polymers [9]. The former includes various aliphatic polyesters derived from renewable resources or produced by fermentation. Notable examples include polylactic acid (PLA), PHA (such as polyhydroxybutyrate, PHB), poly‐(butylene succinate) (PBS), and composite materials based on starch and other natural polysaccharides. These bio‐based, biodegradable polymers exhibit properties analogous to those of conventional plastics, accounting for over 50% of total bioplastics production capacity. Conversely, a substantial proportion (~ 40–45%) of the sector is comprised of ‘drop‐in’ plastics, namely conventional synthetic polymers derived from biological raw materials as opposed to petrochemicals [7]. This category comprises, for instance, bio‐PE (polyethylene derived from bioethanol, chemically identical to conventional polyethylene) and bio‐PET (polyethylene terephthalate partially derived from biomass), as well as biopolyamides and other nonbiodegradable thermoplastics derived from renewable sources. The utilisation of these materials entails a reduced reliance on fossil resources, while maintaining performance that is comparable to their conventional counterparts [10]. Furthermore, these materials require the same recycling channels as traditional fossil‐based plastics [8].

Importantly, PHA have already been produced and deployed on a commercial scale, demonstrating that they are viable industrial bioplastics, not just laboratory‐scale materials. A notable example is Kaneka Corporation in Japan, which produces the PHA copolymer poly(3‐hydroxybutyrate‐co‐3‐hydroxyhexanoate) (PHBH™) on a large scale and markets it under the ‘Kaneka Green Planet’ brand [8]. PHBH™ is produced by microbial fermentation using renewable feedstocks, primarily plant‐derived oils. It has been approved for use in applications involving food contact in multiple jurisdictions, including Japan, the European Union and the United States. Current production capacity has reached the tens‐of‐thousands‐of‐tons‐per‐year scale, with further expansion planned to meet the growing demand for biodegradable packaging materials.

Several other companies have also entered the PHA market at an industrial or preindustrial scale, including Danimer Scientific, CJ Biomaterials, RWDC Industries and TianAn Biologic Materials. This illustrates the rapidly expanding commercial landscape [7]. These producers use a variety of feedstocks, including plant oils, sugars, waste‐derived substrates and methane. This highlights the importance of feedstock selection in determining the economics and sustainability of production. To date, market applications have focused primarily on packaging, food service items, agricultural films and selected biomedical uses, where the combination of biodegradability, biocompatibility and regulatory pressure provides a clear value proposition [9]. At present, the cost of producing PHA remains higher than that of conventional petrochemical plastics, primarily due to the cost of the feedstock, fermentation productivity and the downstream recovery processes. Consequently, the industrial successes to date highlight the importance of process optimisation, the use of low‐cost or waste‐based carbon sources, and application‐driven material design in enabling the economically viable scaling up of production. Existing commercial examples of this provide valuable insights into the opportunities and challenges of large‐scale deployment of PHA as sustainable alternatives within a circular bioeconomy [11].

Although biodegradability is frequently highlighted as a significant benefit of PHA, it is crucial to acknowledge that effective biodegradation hinges heavily on environmental conditions. PHA undergoes rapid mineralisation under industrial composting conditions (50–60 °C with high microbial activity), meeting international standards, such as EN 13432 and ASTM D6400. However, degradation in home compost, soil or marine environments is typically slower and influenced by factors, such as temperature, moisture, the composition of the microbial community, the crystallinity of the polymer and the thickness of the material [11, 12]. Compared with most other biodegradable plastics, PHA also has proven biodegradability in natural environments, including soil and marine settings. This supports their suitability for applications where environmental leakage cannot be fully avoided [9].

Finally, it should be noted that many bioplastics of significant interest, particularly PHA, are produced naturally by microorganisms through specific metabolic pathways. PHA are a class of microbial polyesters that are synthesised and accumulated intracellularly by numerous bacteria as a carbon and energy reserve in conditions of excess carbon substrates and limited other nutrients. This inherent biological capacity offers a significant opportunity to leverage fermentation and metabolic engineering to produce biodegradable plastics from renewable sources efficiently. Consequently, a comprehensive understanding of the bacterial biosynthetic pathways responsible for creating these bioplastics (e.g. the enzymatic mechanisms that lead to the accumulation of PHA in bacteria) is imperative [11]. The next paragraph will therefore examine the microbial metabolic pathways involved in the synthesis of bioplastics, with particular emphasis on biochemical and genetic strategies to optimise bacterial production of PHA and other biodegradable polymers. This integrated approach, at the intersection of industrial microbiology and materials science, is crucial for developing the next generation of sustainable bioplastics and promoting their global dissemination [12].

## Approaches to microbial biopolymer production

Recent advancements in metabolic engineering and synthetic biology have facilitated the microbial production of a broad spectrum of bioplastics via diverse biosynthetic pathways. The categorisation of these pathways can be approached in two broad classifications: the first being the synthesis of the final polymer by the microorganism itself, and the second being the assembly of bio‐based monomers produced via fermentation (Table 1). This distinction is pivotal in comprehending the technological, environmental and economic ramifications of each approach. It is evident that microorganisms, most notably bacteria, possess the capacity to synthesise a diverse array of polymeric materials through highly specific enzymatic pathways. Bacterial strategies for bioplastic production can be categorised into two primary approaches [13]. The first of these is direct polymer synthesis, which manifests as either intracellular accumulation (e.g. polyester granules, such as PHA) or extracellular secretion (e.g. polysaccharides, such as xanthan gum and cellulose). The second approach exploits the microbial fermentation of precursors, whereby bacteria convert renewable substrates into monomeric building blocks [e.g. lactic acid, succinic acid and 1,4‐butanediol (BDO)] that are then polymerised (often chemically) *ex vivo* into polymers, such as PLA or PBS [14]. These biosynthetic pathways are intrinsically linked to fermentation processes, for which the selection of microbial strain, genetic optimisation and strict control of growth parameters (e.g. pH, temperature, nutrient availability and oxygen) are crucial to maximise yield [15, 16].

**Table 1:** Overview of major microbially produced biopolymers, including their chemical class, biosynthetic route, representative producing microorganisms, key properties and typical applications. mcl‐PHA = medium‐chain‐length polyhydroxyalkanoate, PBS = polybutylene succinate, PEF = polyethylene furanoate, PHA = polyhydroxyalkanoate, PHB = poly(3‐hydroxybutyrate), PHBV = (poly(3‐hydroxybutyrate‐co‐3‐hydroxyvalerate)), PLA = polylactic acid, γ‐PGA = poly‐γ‐glutamic acid, ε‐PL = epsilon‐poly‐L‐lysine.

Final product (polymer)	Polymer class	Biological step (what microbes actually do)	Typical producing organism(s)/platform	*Ex vivo* step (if present)	Key properties/typical uses	Reference(s)
PHA (e.g. PHB, PHBV, mcl‐PHA)	Polyester	Cells polymerise hydroxyacyl‐CoA into PHA granules (intracellular accumulation)	*Cupriavidus necator*, *Azohydromonas lata*, *Halomonas* spp., *Pseudomonas* spp.	None (polymer already formed; downstream = recovery/purification)	Fully biodegradable/biocompatible; packaging, agriculture, biomedical	[17, 18]
Bacterial cellulose	Polysaccharide	Cells polymerise glucose → cellulose (extracellular secretion)	*Komagataeibacter* spp.	None	High‐purity hydrogel; food, wound dressings, biomaterials	[99]
Xanthan gum	Polysaccharide	Cells synthesise and secrete xanthan	*Xanthomonas campestris*	None	Thickener/rheology modifier; food/industry	[19]
Alginate	Polysaccharide	Cells synthesise and secrete alginate	*Azotobacter vinelandii* (also *Pseudomonas* spp. in general literature)	None	Viscous gels; encapsulation, biomedical	[100]
γ‐PGA	Polyamide (polyamino acid)	Cells polymerise glutamate → γ‐PGA (capsular/extracellular)	*Bacillus subtilis*	None	Thickener, humectant; cosmetics/food	[101]
ε‐PL	Polyamide (polyamino acid)	Cells polymerise lysine → ε‐PL (extracellular)	*Streptomyces albulus*	None	Antimicrobial; food preservative	[102]
Cyanophycin	Polyamide‐like (poly‐AA)	Cells accumulate polymer intracellularly	Cyanobacteria	None	Nitrogen reserve; potential fertiliser precursor	[103]
PLA	Polyester	Microbes produce lactic acid (LA) (monomer), not PLA	*Lactobacillus* spp. (industrial LA); engineered strains for lactyl‐CoA routes are separate cases	Chemical polymerisation (typically via ring‐opening polymerisation of lactide); enzymatic routes possible in niche cases	Biodegradable; packaging, disposable items, biomedical	[27]
PBS	Polyester	Microbes produce succinic acid and/or BDO (monomers) by fermentation	*Actinobacillus succinogenes*, *Corynebacterium glutamicum* (examples for succinate/BDO supply)	Polycondensation of succinic acid + BDO (chemical catalysis; enzymatic alternatives reported)	Malleable biodegradable; packaging, agricultural films	[32]
PEF	Polyester	Renewable routes supply FDCA + EG (bio‐based monomers; microbial/enzymatic/chemo‐bio routes depending on process)	‘Microbial conversion from sugars/ethanol’ (keep generic unless you specify organism/process)	Chemical polymer synthesis	PET substitute; not readily biodegradable	[33]

### Direct microbial polymer synthesis

It is notable that some polymers are synthesised entirely by microorganisms during the fermentation process, in which the microbes convert substrates into a finished polymer without the need for an external polymerisation step. In such instances, the polymer accumulates within the microbial cells or is secreted into the surrounding medium. A prime example of this is PHA, a class of biodegradable polyesters that are produced by many bacteria as intracellular carbon and energy storage materials. These PHA [e.g. poly(3‐hydroxybutyrate) and its copolymers] are directly polymerised by the bacteria and stored as granules in the cytoplasm. In such instances, enzymes such as polymer synthases have been observed to catalyse the polymerisation of activated monomers *in vivo*, as evidenced by the accumulation of polyhydroxybutyrate (PHB) in *Cupriavidus necator* or the production of cellulose by *Komagataeibacter* [17, 18, 19] (Table 1).

Other examples of polymers biosynthesised by microbes include certain polysaccharides (e.g. bacterial cellulose, xanthan gum and dextran) and polyamides or polyamino acids (e.g. poly‐γ‐glutamic acid produced by *Bacillus species*) (Table 1). In all these cases, the microbial metabolism itself constructs the polymer chain, resulting in a biopolymer that can often be harvested directly from the fermentation broth or cell biomass. This one‐step process, involving only fermentation, has been shown to be advantageous due to the production of the polymer being carried out in a single biological reactor. However, it is important to note that careful optimisation of the culture conditions may be required to achieve maximum polymer yield. For instance, nutrient limitation frequently induces PHA accumulation as a response to stress. Even though microbial synthesis offers greater specificity and operates under milder conditions than petrochemical synthesis, achieving high productivity and titres remains an engineering challenge. Advances in synthetic biology and metabolic engineering are crucial for redirecting metabolic flux and enhancing cell robustness [20]. Additionally, limitations in polymer molecular weight or composition that are beyond the cells' capabilities may be present. PHA are a family of microbial‐derived polyesters that have gained considerable interest as fully biodegradable bioplastics. These polymers serve as carbon and energy reserves in numerous bacteria, accumulating as insoluble granules within the cytoplasm [21]. It is estimated that over 150 types of PHA monomers have been identified, resulting in a range of final polymer properties, including rigid thermoplastics (e.g. PHB) and elastomers, with PHB exhibiting mechanical properties comparable to those of polypropylene (PP) [22]. The appeal of these materials lies in their biodegradability (mediated by PHA depolymerases in various natural environments) and biocompatibility. The biosynthesis of PHB, the most common PHA, is catalysed by three key enzymes: β‐ketothiolase, acetoacetyl‐CoA reductase, and, crucially, PHA synthase (encoded by the *phaC* gene), which polymerises 3‐hydroxyacyl‐CoA substrates. The specificity of these synthases determines the length of the polymer chain [23]. For example, *Cupriavidus necator* mainly produces PHB (C4 monomers), while *Pseudomonas putida* can accumulate medium‐chain PHA from fatty acids, exploiting β‐oxidation. In these producers, MCL‐PHA composition is strongly influenced by the supplied carbon source (e.g. fatty acids vs sugars) and the activated metabolic routes (β‐oxidation vs *de novo* fatty‐acid synthesis), which determine the pool of (R)‐3‐hydroxyacyl‐CoA monomers available to PhaC. PHA accumulation is typically induced by nutritional limitations (e.g. nitrogen or phosphorus deficiency) in the presence of excess carbon [21].

It is estimated that over 300 bacterial species can produce PHA. Examples of such organisms include *Azohydromonas lata* and *Cupriavidus necator*, which have been shown to accumulate the polymer up to 80–90% of their dry cell weight [21, 24]. The optimisation process, particularly in high‐cell‐density fed‐batch cultures, has resulted in titres approaching 100 g·L^−1^ in bioreactors. To reduce costs and enhance sustainability, research is centring on the utilisation of extremophiles (e.g. *Halomonas* spp.), which facilitate fermentation under nonsterile conditions and streamline extraction through salt‐induced cell lysis [25, 26].

### Fermentation‐derived monomers and postpolymerisation

A significant number of bio‐based polymers are synthesised via a two‐step process. Firstly, microorganisms ferment renewable raw materials to produce monomeric precursors. Secondly, these monomers are polymerised through chemical or enzymatic reactions outside the cell. In this approach, the role of the microbe is to efficiently generate building blocks (monomers, such as organic acids and diols or diamines), rather than the polymer itself. A notable example is PLA. In this process, lactic acid is initially produced on a large scale through bacterial fermentation of sugars, employing strains of Lactobacillus as a model. This biologically derived lactic acid, predominantly in the L‐isomer form, is subsequently converted chemically to lactide *ex vivo* and then polymerised, frequently via ring‐opening polymerisation, to yield the PLA polymer. It is important to note that, in the conventional industrial route, microorganisms do not synthesise PLA directly; rather, they supply lactic acid that is subsequently polymerised in an industrial reactor. Despite the established effectiveness of this approach, recent advances in metabolic engineering have paved the way for direct microbial synthesis of PLA [27]. This ‘one‐pot’ strategy involves the expression of key enzymes (e.g. propionyl‐CoA transferase and PHA synthase) in engineered bacterial strains (e.g. *E. coli*), enabling the conversion of intracellular lactic acid into lactyl‐CoA and its direct polymerisation into PLA chains or hybrid copolymers [e.g. poly (lactic acid‐co‐3‐hydroxybutyrate)], thus bypassing intermediate chemical and purification steps. In addition to PLA, bacteria play a pivotal role in the supply chain for bio‐derived monomers used to produce other polyesters. Notably, as a biodegradable polyester, PLA can also undergo enzymatic depolymerisation [28], and recent work has shown that tailored combinations of hydrolytic and oxidative enzymes can markedly accelerate its breakdown under mild conditions [29], offering promising biocatalytic routes for efficient PLA end‐of‐life recycling.

In a similar manner, succinic acid, 1,3‐propanediol, adipic acid, ethylene glycol and other molecules can be fermented by engineered microbes from renewable feedstocks. These molecules serve as precursors for synthesising polymers like polyesters or polyamides through traditional polymer chemistry. For instance, the combination of bio‐based succinic acid and BDO has been demonstrated to yield poly(butylene succinate), while bio‐based 1,3‐propanediol has been utilised in the production of poly(trimethylene terephthalate). PBS is synthesised by polycondensation of succinic acid and BDO. These precursors can be obtained through the fermentation process [30, 31]. Microbial strains such as *Actinobacillus succinogenes* and engineered *Corynebacterium glutamicum* have been shown to produce high titres of succinic acid (above 100 g·L^−1^), and BDO can also be bio‐produced from glucose [32]. The mixture of bio‐succinate and bio‐BDO facilitates the synthesis of a predominantly bio‐based PBS, a malleable, biodegradable polymer used in the manufacture of agricultural films and packaging. A similar principle applies to the new‐generation polyester poly(ethylene furanoate) (PEF), a potential substitute for PET, whose monomers, 2,5‐furandicarboxylic acid (FDCA) and ethylene glycol, are derived from microbial or enzymatic processes starting from sugars and bioethanol. However, PEF is not readily biodegradable [33].

Enzymatic polymerisation methods can also be employed at this second step, for instance by using lipase enzymes as catalysts for polyester formation, offering a milder alternative to chemical catalysts in some cases.

## Properties and structural features of PHA‐based biopolymers

PHA are biodegradable, biocompatible, nontoxic thermoplastics, insoluble in water and soluble in various chlorinated solvents. PHA were first discovered in 1926 by the French researcher Maurice Lemoigne, who identified them as intracellular inclusion bodies in *Azotobacter chroococcum* and *Bacillus megaterium*. This polymer was later characterised as PHB [34]. PHA are composed of linear hydroxy fatty‐acid monomers. The length and structure of the side chain (R group) in each monomer strongly influence the overall structure and physicochemical properties of the resulting polymer [35, 36, 37]. When the monomers contain 3–5 carbon atoms, the resulting polymer is classified as a short‐chain‐length PHA (SCL‐PHA). These polymers resemble conventional thermoplastics and are typically brittle and stiff, exhibiting a high degree of crystallinity (60–80%). Medium‐chain‐length PHA (MCL‐PHA), composed of monomers with 6–14 carbon atoms, display properties closer to elastomers [38]. Polymers with monomers containing more than 14 carbon atoms are referred to as long‐chain‐length PHA (LCL‐PHA), (Table 2). MCL‐PHA and LCL‐PHA generally have a low degree of crystallinity (around 25%), high elongation at break and low melting temperatures [39, 40]. SCL‐PHA are predominantly composed of PHB, a stiff and brittle polymer with a melting temperature (*T*_m_) of approximately 179 °C. This value lies close to its decomposition temperature (200–300 °C) [41, 42], which limits its thermal processing window. A common strategy to enhance the thermal and mechanical properties of PHA is the biosynthesis of random copolymers, such as poly(3‐hydroxybutyrate‐co‐3‐hydroxyvalerate) (PHBV), PHBH and the terpolymer poly(3‐hydroxybutyrate‐co‐4‐hydroxybutyrate‐co‐3‐hydroxyvalerate) (P(3HB‐4HB)‐3HV) [40, 43]. PHA can also be classified according to the carbon source used in their production. The first class is produced from traditional carbon sources, such as sugars, vegetable oils and animal fatty acids, which contain the hydroxy acids forming the polymeric chains of PHA [44]. Additionally, a second class is derived from the use of modified carbon sources, allowing the creation of new polymers with different functional groups on their side chains and with altered morphology and physical properties [45]. Carboxylation, hydroxylation and graft copolymerisation can modify their structure to fine‐tune key properties, such as hydrophobicity and biocompatibility, rendering these polymers more versatile for a broad spectrum of applications [46].

**Table 2:** Overview of structurally characterised PHA polymers and their properties. PHB = poly3‐hydroxybutyrate, PHBV = poly(3‐hydroxybutyrate‐co‐3‐hydroxyvalerate), PHBH = poly(3‐hydroxybutyrate‐co‐3‐hydroxyhexanoate).

PHA typologies	Chain length	Morphology	Thermal properties	Mechanical properties	Reference(s)
PHB	Short (C4)	α/β‐crystal, spherulite, shish–kebab	High melting point (170–180 °C)	High strength (30–40 MPa), high hardness, high brittleness, low elongation at break	[43]
PHBV	Short–medium (C4–C5)	Semicrystalline/amorphous depending on 3HV content	Degradation in single step (nonradical random cleavage process)	High 3HV content confers higher flexibility but low tensile strength and modulus	[59, 60]
PHBH	Medium (C6–C14)	Random/block copolymers	The Increase in 3HH comonomer content significantly reduces the *T*_m_	High 3HH increases the elongation at break and decreases the strength and modulus	[61, 62]

PHA can have different crystal morphologies. In 1997, Yoshiharu *et al*., obtained a single crystal of PHB from a mixed chloroform‐methanol solution (1/7) [47]. The most common crystal form is spherulite. These structures are commonly formed when PHA are rapidly cooled from the melt or precipitated from a concentrated solution. This type of crystallisation occurs via primary nucleation to form a spherulite core, followed by the radial growth of fibrillar crystals at a constant rate [40]. Moreover, the increase of the crystallisation temperature leads to a change in the morphology of PHA crystal between banded spherulites and nonbanded spherulites [48]. A third PHA morphology reported in the literature is the shish–kebab structure, which can be obtained by continuously stirring a dilute polymer solution. Although the exact mechanism underlying its formation is not yet fully understood, it is thought to be related to the coil–stretch transition mechanism [49]. Masahiro and co‐workers observed the shish–kebab morphology in ultra‐thin PHB films prepared by dissolving ultrahigh‐molecular‐weight PHB in chloroform solution [50]. Bacterial PHB can crystallise in two forms, the *a*‐ and *b*‐crystal. The most common crystal structure of PHB is the α‐form, which is generally produced by controlled melt, cold or solution crystallisation. The *β*‐form of PHB can be created by changing the orientation of free chains or tie chains in the amorphous regions between the *a*‐form lamellar crystals, or from structural modifications of the *α*‐crystal into *β*‐crystal. Due to its architecture, the *β*‐structure has an improved mechanical strength, and the properties of the crystal can remain stable for months, thanks to the inhibition of the secondary crystallisation process [51].

The properties of a PHA polymer can be influenced by several factors, such as the polarity, the geometry and the stereochemistry. The chemical and physical properties are strictly linked to the polymer architecture, and small changes in it can have a deep impact on the overall characteristics and behaviour. In recent years, the mechanical and thermal properties of PHA have been well described, highlighting that they are comparable to those of conventional petroleum‐based plastics, such as PET, PP and PE [52, 53]. It is known that PHA exhibits low thermal stability at temperatures over 170 °C and PHB has very strict mechanical properties strongly influenced by nucleation density and crystallisation rate. Thus, it is possible to improve PHA properties by the introduction of comonomer units and the formation of copolymeric materials, such as PHBV, P3HB4HB and PHBHHx. In this way, it is possible to improve the elongation to break and toughness of the resulting polymer film at a cost of lower strength and stiffness [54]. The main properties of a PHA polymer are also influenced by their chains molecular weight. It was reported that PHA shows better mechanical properties when the chains molecular weight is above 400 kDa, and for thermoplastic applications the Mw should be higher than 600 kDa [55, 56]. A study by Luo *et al*., [57] demonstrated that different properties of PHBV copolymers, such as the melting temperature, the lamellar and amorphous thickness and the tensile strength increased proportionally to the increasing of the molecular weight of the crystals.

Among PHA typologies, PHB represents the most common biological polyester, and its crystal structure has been solved by X‐ray studies on oriented fibres (Table 2). The fibre diagram indicated a repeat along the chain axis of 0.596 nm, corresponding to the length of two residues with two antiparallel chains packed in an orthorhombic unit cell [43]. Conformational analysis based on intramolecular energy calculations has shown that the polymer chain adopts a left‐handed helical conformation. PHB typically forms lath‐shaped crystals with approximate dimensions of 0.3–2 μm along the short axis and 5–10 μm along the long axis. The thickness of these monolamellar single crystals ranges from 4 to 10 nm, depending on factors such as molecular weight, solvent and crystallisation temperature. Researchers found that some bacteria (such as *Ralstonia eutropha*) were able to produce not only hydroxybutyrate units but also 3‐hydroxyvalerate (3HV) when supplemented with glucose and propionate [58]. In this way, the copolymer PHBV was produced, demonstrating enhanced flexibility compared with PHB (Table 2). Indeed, by adjusting the ratio of 3HB to 3HV monomers, the polymer properties can be fine‐tuned to produce either flexible films or rigid objects [59, 60]. PHBV is synthesised from two monomers: 3‐hydroxybutyrate (3HB) and 3HV. A change in the ratio of the two monomers can significantly modify the mechanical and thermal characteristics of the polymer. Poly(3‐hydroxybutyrate‐co‐3‐hydroxyhexanoate) is a random copolymer composed of 3‐HB and 3‐hydroxyhexanoate (3‐HH) units. A higher mole percentage of 3‐HH segments leads to lower melting temperatures, reduced crystallinity and diminished storage modulus and overall strength, whereas PHBH copolymers with a lower 3‐HH content tend to experience physical ageing over time, resulting in an increase in modulus and strength but a loss in ductility [61]. PHBH can occur in both random (most employed) and block configurations. While pure PHB displays a single melting point around 170 °C, random PHBH copolymers with different 3‐HH concentrations may exhibit two nearby melting peaks at lower temperatures. As the proportion of 3‐HH comonomer rises, the glass transition temperature (*T*_g_) is suppressed, and the *T*_m_ and the final degree of crystallinity are notably reduced (Table 2). PHBH samples may also undergo secondary crystallisation at room temperature due to ageing, a phenomenon often described as cold crystallisation. Furthermore, the thermal degradation temperature of PHBH copolymers is strongly influenced by the 3‐HH content: increasing the amount of 3‐HH enhances elongation at break but reduces tensile strength and modulus because of the greater flexibility imparted by the 3‐HH segments [62].

## *In vitro* PHA biosynthesis: enzymes, variants and reaction platforms

Advances in biotechnology have increasingly focused on mitigating the environmental impact of conventional plastics, resulting in technological breakthroughs that enable the industrial‐scale biosynthesis of bio‐based monomers from renewable carbon sources. To develop the next generation of bio‐based polyesters, research must not only ensure sustainability but also achieve enhanced competitiveness through optimised technological performance and improved functional characteristics [63].

The *in vitro* synthesis of PHA has emerged as a promising alternative to traditional microbial fermentation, aiming to overcome limitations in cellular metabolism, product recovery and process control. Reconstructing PHA biosynthetic pathways outside living cells offers the opportunity to design fully controllable, modular and cell‐free systems capable of achieving higher yields and tailored polymer compositions. The production cost of PHA remains significantly higher than that of conventional petrochemical plastics. Thus, the development of more efficient and economically viable production processes is of paramount importance. Cell‐free metabolic engineering (CFME) has already demonstrated great potential in optimising a variety of biosynthetic pathways, suggesting that analogous cell‐free systems could be strategically exploited to enhance PHA biosynthetic efficiency and yield [64]. The *in vitro* enzyme‐catalysed synthesis of polyesters is an emerging approach that has been actively pursued in the last decade and offers an eco‐friendly alternative to conventional chemical polymerisation methods, providing several advantages in terms of efficiency, sustainability and reaction conditions. Enzymatic polymerisation is an *in vitro* process that occurs via nonbiosynthetic pathways and is catalysed by an isolated enzyme. In direct enzymatic polymerisation, the purified PHA synthase (PhaC) catalyses the polymerisation of the activated substrate, hydroxyacyl‐coenzyme A (R)‐HA‐CoA, to PHA. PhaC enzymes have been categorised into four major classes based on their primary sequence, substrate specificity and subunit composition [65]. Class I, Class III and Class IV produce SCL‐PHA depending on propionate, butyrate, valerate and hexanoate precursors, while Class II PhaC synthesise MCL‐PHA based on the alkane (C6–C14) precursors [66].

**Fig. 2:**
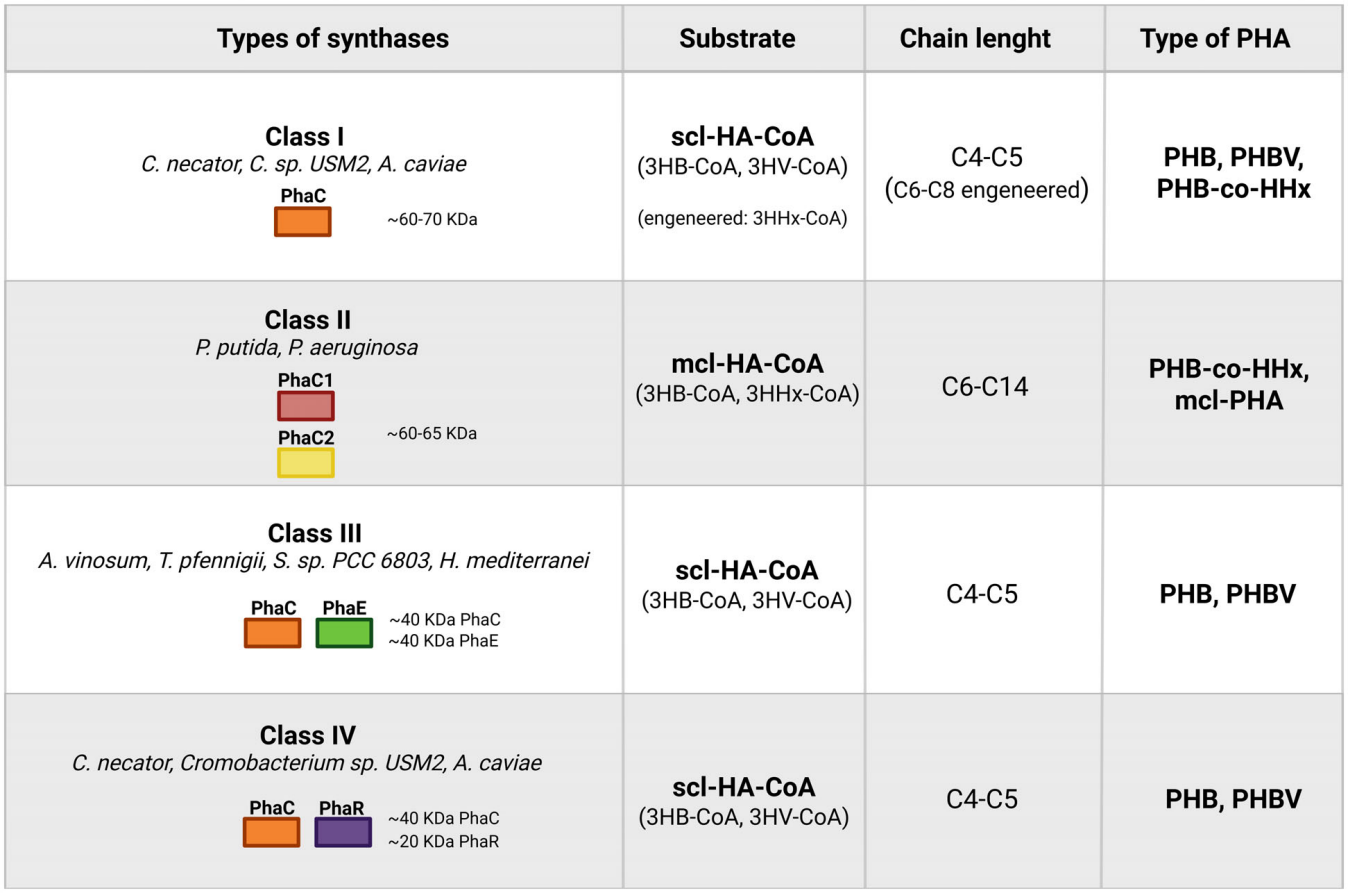
Classification of polyhydroxyalkanoate (PHA) synthases. Schematic representation of the different classes of PHA synthases involved in PHA biosynthesis, their associated substrates and the carbon‐chain length of the monomer units forming the final polymer [66, 67, 68, 69, 72, 73, 75, 80, 81]. 3HV‐CoA = 3‐hydroxyvaleryl‐coenzyme A, 3HB‐CoA = 3‐hydroxybutyryl‐coenzyme A, 3HHx‐CoA = 3‐hydroxyhexanoyl‐coenzyme A, kDa = kilodaltonmcl‐HA‐CoA = medium‐chain‐length hydroxyacyl‐coenzyme A, mcl‐PHA = medium‐chain‐length polyhydroxyalkanoate, PHB = poly(3‐hydroxybutyrate), PHB‐co‐HHx = poly(3‐hydroxybutyrate‐co‐3‐hydroxyhexanoate), PHBV = poly(3‐hydroxybutyrate‐co‐3‐hydroxyvalerate), scl‐HA‐CoA = short‐chain‐length hydroxyacyl‐coenzyme A. PhaC, PhaE and PhaR denote distinct synthase subunits [66, 71, 73, 80, 81].

Class I PhaC function as homodimers composed of two identical PhaC subunits and preferentially use SCL hydroxyacyl‐CoA substrates such as 3‐hydroxybutyryl‐CoA (C3–C5) to synthesise polymers like PHB and its copolymers. PhaC from *Cupriavidus necator* [67] (ex *Ralstonia eutropha*)‐PhaC_Re/Cn, *Chromobacterium* sp. *USM2*—PhaC_Cs and *Aeromonas caviae*—PhaC_Ac belong to this class [68, 69]. The activity of these enzymes was tested *in vitro* and the specific activity towards the polymerisation of 3HB‐CoA was evaluated. In terms of specific activity, PhaC_Cs shows higher catalytic rates than PhaC_Cn (about 5‐fold greater in comparable tests) [70]. Regarding substrate specificity, PhaC_Ac is notable because engineered variants increase the proportion of the C6 monomer (3HHx) in copolymers [71]. The regulatory role of accessory proteins on PHA synthase activity was also investigated for PhaC_Ac and PhaC_Re. The activity of PhaC_Ac was markedly enhanced in the presence of phasins (PhaP), which modulate both enzyme activity and polymer granule formation. In contrast, no activation effect was observed for PhaC_Re. Phasins specifically interact with certain PhaC enzymes, improving polymer chain elongation and release and playing an essential regulatory role in PHA biosynthesis [69] (Fig. 2).

Class II PhaC enzymes are PHA synthases specialised in the production MCL‐PHA, typically containing monomers with 6–14 carbon atoms (e.g. 3‐HH, 3‐hydroxyoctanoate and 3‐hydroxydecanoate). Since type II enzymes are mainly found in *Pseudomonas*, the most *in vitro* studied PhaC that belong to this class are from *Pseudomonas putida* (GPo1) PhaC1/PhaC2 and *Pseudomonas aeruginosa*—PhaC1/PhaC2 [72, 73, 74]. *Pseudomonas putida* PhaC1 is the major active enzyme, showing *in vitro V*_max_ ~ 40 μmol·min^−1^ and *K*_M_ ~ 125 μm on (R)‐3‐hydroxyoctanoyl‐CoA; PhaC2 has lower turnover (~ 2–3 μmol·min^−1^) but higher affinity [66, 74]. These enzymes strongly prefer C6–C10 substrates, with highest activity on C8–C10 (3HO, 3HD‐CoA). Purified soluble forms rapidly lose activity, whereas granule‐bound enzymes remain catalytically competent. *Pseudomonas aeruginosa* PhaC1/2 display a similar MCL specificity but little or no activity on short‐chain 3HB‐CoA [73]. The *in vitro* activity of these enzymes is mostly detected in crude extracts, confirming dependence on granule context for stability. Both systems show a lag phase typical of Class II PhaCs, requiring priming and CoA recycling. In both cases, the resulting polymers are amorphous and elastomeric, unlike the crystalline PHB from Class I enzymes. Together, they define the biochemical standard for studying Class II PHA synthases *in vitro* (Fig. 2).

Class III PHA synthases are heterodimeric enzymes composed of PhaC (catalytic) and PhaE (structural) subunits, both required for activity [66]. They are mainly found in photosynthetic and halophilic bacteria, such as *Allochromatium vinosum*, *Thiocapsa pfennigii*, *Synechocystis* sp. *PCC 6803* and *Haloferax mediterranei*. Unlike Class II enzymes, which synthesise SCL‐PHA, primarily PHB or PHBV, Class I enzymes use (R)‐3HB‐CoA and (R)‐3HV‐CoA as substrates. The best characterised *in vitro* model is PhaEC from *Allochromatium vinosum* [75]. Activity is completely lost if PhaE is omitted, confirming the strict interdependence of the two subunits. *Thiocapsa pfennigii*, *Synechocystis* and *Haloferax mediterranei* enzymes show similar *in vitro* behaviour [76, 77, 78]. Class III synthases thus define a unique PHA biosynthetic mechanism combining catalytic and structural regulation [79] (Fig. 2).

Finally, Class IV PhaC synthases are heterodimeric enzymes composed of PhaC (catalytic subunit) and PhaR (regulatory/accessory subunit). They are mainly found in Gram‐positive *Bacillus* species, such as *Bacillus megaterium* and *Bacillus cereus* [80, 81]. The enzyme catalyses the polymerisation of SCL 3‐hydroxyacyl‐CoAs (C3–C5), producing PHB or PHBV polymers. Class IV enzymes are active as αβ heterodimers, unlike Class I monomers or Class II homodimers. Their catalytic mechanism involves sequential transesterification of (R)‐3‐hydroxyacyl‐CoA thioesters. In Class IV PHA synthases, PhaR is an integral catalytic partner, not a regulatory one [82]. It is required to fold, stabilise and anchor the PhaC catalytic core, forming a functional heterodimer that can polymerise (R)‐3HB‐CoA into PHB. Without PhaR, *in vitro* assays show no detectable activity, confirming that PhaR is indispensable for Class IV PhaC function (Fig. 2).

Although conceptually straightforward, *in vitro* systems are limited by the high cost and instability of CoA‐activated substrates that restricts their scalability. Engineered PhaC are essential biocatalysts for creating custom PHA polymers in cell‐free (*in vitro*) systems, overcoming the metabolic constraints and substrate limitations of native microbial hosts. The primary functional element in all PhaC enzymes is a conserved α/β‐hydrolase domain containing the catalytic triad, typically cysteine (Cys), aspartate (Asp) and histidine (His) often embedded within a characteristic PhaC box sequence ([GS]‐X‐C‐X‐[GA]‐G) [83]. The catalytic cysteine residue acts as the nucleophile, forming a covalent thioester intermediate with the hydroxyacyl‐CoA monomer. Engineering novel PhaC variants is therefore crucial for advancing PHA technology. Such variants enable precise control over monomer composition, for example, tuning the ratio of 3HB to 3HV or incorporating non‐native monomers, such as lactate (LA), to modulate the polymer's thermal, mechanical and degradation properties. Moreover, engineered PhaC enzymes are essential for producing advanced polymer architectures, including well‐defined block copolymers and highly specific random copolymers, which are not achievable with native enzymes. Engineering can also focus on improving the enzyme's intrinsic properties, such as increasing its catalytic efficiency (*k*_cat_) and enhancing thermostability for robust *in vitro* synthesis. Finally, variants allow researchers to control chain termination, thereby precisely adjusting the final molecular weight and polydispersity of the resulting polymer. Engineered PhaC variants exemplify the growing precision with which PHA synthases can be tailored for *in vitro* biopolymer synthesis (Table 3). The PhaC (Ac) NSDG mutant (N149S, D171G from *Aeromonas caviae*) significantly enhances 3HHx incorporation, expanding monomer diversity and enabling the formation of more flexible copolymers [84]. The Lactate‐Polymerising Enzyme (LPE), such as PhaC1 (Ps6‐19) from *Pseudomonas* sp. 61–3, catalyses the *in vitro* polymerisation of lactyl‐CoA, facilitating the enzymatic synthesis of PLA and related LA‐copolymers [85]. The sequence‐regulating PhaC (AR) from *Allochromatium vinosum* and *Ralstonia eutropha* introduces precise control over polymer microstructure, enabling the synthesis of block copolymers, such as P(3HB)‐co‐P(3 HV) with defined sequence and segment length [86]. Finally, the thermostable PhaC1 (SG) (STQK) variant from *Pseudomonas* sp. *SG4502* incorporates a modified STQK loop, conferring stability and sustained catalytic activity under elevated‐temperature *in vitro* conditions [87]. Together, these engineered enzymes provide a molecular toolkit for precisely tuning polymerisation kinetics, monomer composition and chain length, effectively transforming PHA synthesis from a natural metabolic process into a programmable enzymatic platform for sustainable, cell‐free biopolymer production.

**Table 3:** Overview of engineered PhaC variants, including the specific mutations, source microorganism and *in vitro* function. 3HHx = 3‐hydroxyhexanoate, LA = lactate, LPE = lactate‐polymerising enzyme, P(3HV) = poly(3‐hydroxyvalerate), P(3HB‐co‐3HHx) = poly(3‐hydroxybutyrate‐co‐3‐hydroxyhexanoate), P(LA‐co‐3HB) = poly(lactate‐co‐3‐hydroxybutyrate), PHA = polyhydroxyalkanoate, PhaC = PHA synthase, PLA = polylactic acid.

PhaC variant name	Mutation(s) and source	Primary *in vitro* function	Reference(s)
PhaC Ac NSDG	N149S, D171G (*A. caviae*)	Significantly increases the fraction of 3‐hydroxyhexanoate (3HHx) in the P(3HB‐co‐3HHx) copolymer	[84]
PhaC1 Ps6–19 (LPE)	Evolved/Multiple mutations (*Pseudomonas* sp. 61–3)	A highly utilised Lactate‐Polymerising Enzyme (LPE), enabling the biosynthesis of Polylactic Acid (PLA) or LA‐copolymers (P(LA‐co‐3HB))	[85]
PhaC AR	Multiple mutations (*A. vinosum*, Class III)	Engineered for sequence regulation, capable of synthesising defined PHA architectures like block copolymers (e.g. P(3HB) − co − P(3HV) by controlling monomer insertion)	[86]
PhaC1 (SG) (STQK)	S324T, Q480K (*Pseudomonas* sp. SG4502)	Enhanced thermostability and LA polymerising activity, ideal for high‐temperature, sustained *in vitro* synthesis	[87]

A significant leap forward has been achieved with CFME platforms. These systems integrate up to 14 enzymes within a single reaction mixture, including modules for energy and cofactor regeneration (e.g. NADPH recycling), allowing the conversion of low‐cost substrates like glucose or maltodextrin directly into PHB without external ATP supplementation. This ‘one‐pot’ strategy has recently demonstrated theoretical yields exceeding 90%, marking an important step towards industrial viability [88]. In parallel, hybrid chemo‐enzymatic systems have been proposed to combine chemical activation of hydroxy acids with enzymatic polymerisation by PhaC, providing flexibility in substrate selection and facilitating the synthesis of copolymers, such as P(3HB‐co‐3HV). Additional innovations include enzyme immobilisation on solid supports to improve stability and reusability, synthetic or electrochemically regenerated cofactors to lower operating costs, and artificial PhaC mimics designed through protein engineering or synthetic chemistry [64].

Since the extraction of intracellular PHA generally requires cell disruption, which is time‐consuming, relies on harsh chemical treatments and necessitates the re‐cultivation of new bacterial cells for subsequent production cycles, another promising direction to enhance *in vitro* PHA biosynthesis involves the use of enzyme surface‐display systems, which spatially organise catalytic cascades on solid or biological surfaces. In this approach, enzymes such as PhaA, PhaB and PhaC are anchored onto carrier matrices, protein scaffolds or microbial cell surfaces via engineered linkers or anchoring domains. By confining sequential enzymes within nanometre‐scale proximity, these assemblies promote substrate channelling, minimise diffusion losses and substantially increase the catalytic efficiency of multi‐step pathways. Surface display can be implemented using cell‐free scaffolding proteins (e.g. SpyTag/SpyCatcher systems) or via bacterial outer‐membrane anchoring in semi‐cellular systems. In the context of PHA synthesis, co‐displaying PhaC with its upstream enzymes accelerates polymer chain elongation and enhances monomer conversion rates. When integrated with CFME or immobilised enzyme technologies, surface display offers a powerful route towards compact, reusable and spatially organised biocatalytic systems, bridging the gap between free‐solution enzymatic reactions and structured bioreactors [89].

The growing development of *in vitro* methodologies for PHA synthesis is fundamentally reshaping the way bioplastics are produced. By moving beyond cell‐dependent fermentation, researchers are building modular, programmable enzymatic systems that enable precise control over every stage of polymer formation, from monomer selection to chain architecture. This shift marks the beginning of a new era in biopolymer engineering, where efficiency and environmental responsibility can coexist within a single platform. Ultimately, these advances open the door to next‐generation biomanufacturing processes capable of producing custom‐designed biodegradable materials that are both economically viable and environmentally sustainable.

Despite these promising solutions, the limited commercial availability of biopolymers is mainly due to a combination of technological, economic and infrastructural constraints. In fact, technologies for the production and purification of bio‐based monomers are not yet fully mature. Many bio‐derived building blocks are obtained through fermentation or complex biomass conversion processes, which often result in low yields and challenging downstream purification steps. Achieving polymer‐grade purity requires energy‐intensive and costly separation technologies, making large‐scale implementation difficult.

The raw materials used to produce bio‐based monomers frequently compete with food and feed resources. While PHA offers a sustainable alternative to conventional plastics, their production comes with challenges that impact economic viability. Many current processes rely on first‐generation biomass, such as sugars, corn or vegetable oils. This creates concerns regarding land use, food security and sustainability, especially when agricultural resources are allocated to industrial purposes rather than food production. Moreover, the high production cost of purified enzymes and their substrates for the *in vitro* synthesis of PHA could limit the large‐scale adoption of these methods. The commercialisation of PHA remains constrained by their significantly higher production costs compared with petroleum‐based plastics [90]. The price of polypropylene and polyethylene is about US $1.25–2.53 per kg, while that for PHA has been reported to be up to 16 times higher than the major petroleum‐derived polymers [91, 92]. The overall production costs of bio‐based monomers and their subsequent polymerisation into bio‐based polyesters remain relatively high compared with their fossil‐based counterparts and contribute to this economic gap. Research is shifting towards cost‐reduction strategies in order to increase the competitiveness of PHA, including the introduction of the developed *in vitro* solutions previously described into already existing processes to enhance their productivity and allow the scale‐up of these high‐cost technologies and to improve their sustainability [93].

Another thing that must be taken into consideration is that the widespread adoption of PHA will depend strongly on the development of international standards for biodegradable plastics, along with improvements in waste management infrastructure. Clear global certification frameworks for biodegradability and compostability are essential to ensure consistent material performance, avoid misleading environmental claims, and build trust among consumers, manufacturers and regulators. Without appropriate disposal and treatment pathways, even biodegradable materials may fail to deliver their intended sustainability advantages [90]. Taking this into consideration, coupling PHA synthesis with waste valorisation strategies can enhance both economic feasibility and environmental performance.

## Future perspectives in enzymatic bioplastic production

Despite significant progress in microbial and enzymatic PHA production, the translation of these technologies to industrial‐scale processes is still limited by specific technical and economic constraints. Notably, the high cost of CoA‐activated substrates, the limited long‐term stability and turnover of PHA synthases in cell‐free systems and the absence of scalable reactor concepts for multi‐enzyme cascades are the main factors hindering industrial implementation. Furthermore, downstream processing and polymer recovery continue to be significant cost drivers, even for advanced enzymatic or hybrid production platforms. Addressing these challenges will require integrated advances in areas, such as enzyme engineering, cofactor regeneration, process intensification and reactor design, rather than incremental improvements at the level of individual enzymes.

The future of enzymatic bioplastic production will depend on the effective integration of synthetic biology, metabolic engineering and enzyme design to overcome current limitations in enzyme stability, substrate availability and process scalability. The convergence of these disciplines facilitates the construction of novel biosynthetic pathways and the development of tailored enzymes, thereby expanding the diversity of PHA and increasing process yields. Innovations such as cell‐free multi‐enzymatic systems and enzyme surface display have been shown to help overcome limitations associated with intracellular processes, thereby enhancing catalytic efficiency, stability and control over polymer composition [94].

Furthermore, it is imperative to emphasise the importance of enhancing substrate flexibility to ensure the continuity of sustainable production. The use of engineered microbes to convert alternative feedstocks, including but not limited to CO_2_, CH_4_ and lignocellulosic biomass, has been demonstrated to be a viable solution to the current problems of reliance on food‐based resources and high production costs. In conjunction with advancements in continuous and high‐cell‐density fermentation, as well as the utilisation of extremophiles, these strategies facilitate the development of robust, scalable and less resource‐intensive bioprocesses [95, 96].

Notwithstanding these innovations, economic viability remains a considerable challenge. The present cost of producing PHA exceeds that of petrochemical plastics, primarily due to the expense of feedstocks and the complexity of recovery methods. Consequently, enhancing strain robustness, optimising bioreactor efficiency and streamlining downstream processing are pivotal to attaining industrial competitiveness. Sustainability assessments, including life‐cycle analyses (LCAs), are mandatory for validating the environmental benefits of emerging technologies, particularly in terms of carbon footprint and biodegradability [97].

Finally, it must be emphasised the pivotal role of policy and regulatory support in facilitating market adoption. This encompasses the provision of financial incentives, the establishment of clear biodegradability standards and the implementation of policies that favour bio‐based alternatives over fossil‐derived plastics. The acceleration of scale‐up and commercialisation is further facilitated by collaboration between the public and private sectors [98]. Finally, enzymatic PHA production will only become economically competitive with established fermentation‐based routes if there is an improvement in biocatalyst performance, cost‐effective cofactor management and scalable process design. The integration of scientific innovation with economic and policy frameworks is pivotal in the evolution of enzymatic bioplastics from niche materials to foundational elements of a circular bioeconomy.

## Conflict of interest

The authors declare no conflict of interest.

## Author contributions

SF conceived the review topic and structure. GG, LB and EP collected and curated the data. GG, LB and EP drafted the original manuscript, with MA and SF contributions. GG, MA and SF reviewed and edited the manuscript. SF supervised the work and secured funding. All authors read and approved the final version of the manuscript.
